# Compromise revisited: inhibitory synapse and electrical coupling effects on bilateral phasing in the leech heartbeat system

**DOI:** 10.1186/1471-2202-13-S1-P161

**Published:** 2012-07-16

**Authors:** Adam L Weaver, Kristen B Cowens

**Affiliations:** 1 Department of Biology, Saint Michael's College, Colchester, VT 05439, USA

## 

The leech heartbeat central pattern generator (CPG) consists of a network of heart interneurons (HN) that coordinate heart excitor (HE) motor neuron activity via inhibitory chemical synapses. Each segmental pair of HE’s is connected to one another via electrical coupling. Depending on the segment, the pair of motor neurons in the living system is active across a wide range of phase differences from nearly in-phase to anti-phase [1]. Prior efforts to model this complete network have not quantitatively matched the intersegmental phase differences observed [2]. We have created a reduced network model in Simulink to explore parameters that contribute to these phase differences.

In our network model, we implemented known neuronal properties and synaptic connections from a single segmental ganglion as shown in Figure 1. In our initial model run, the HN’s were modeled as endogenous bursters as previously described [3]; the HE’s were modeled as tonic firers. We varied three parameters in this study: phased delay of the right HN synaptic input (*Φ_Syn_*) and the maximum conductances of the inhibitory synapse (*g_Syn_*) and electrical coupling (*g_coup_*).

**Figure 1:**
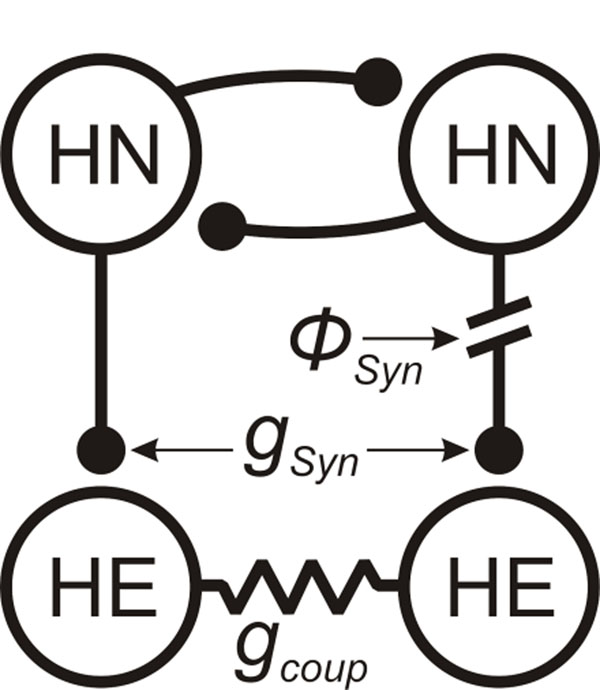
Leech heartbeat circuit diagram showing identified heart interneurons (HN) of the CPG and their pattern of inhibitory synaptic connections (lines with circles) onto each other and the heart excitor motor neurons (HE). The resistor symbol indicates rectifying electrical coupling connecting two HE neurons in the same segment. Three parameters were varied in this study: the phase of the right HN synaptic input (*Φ_Syn_*) and the maximum conductances of the inhibitory synapse (*g_Syn_*) and electrical coupling (*g_coup_*).

We found that in this network *g_Syn_* must be at least 150 times greater than *g_coup_* in order to obtain 1:1 entrainment of the HE’s with the HN’s. Under conditions with zero *Φ_Syn_*, increased *g_coup_* led to increased instantaneous spike frequencies (ISF) and reduced duty cycle, primarily due to a delayed burst beginning. Increasing *g_Syn_* alone with zero *Φ_Syn_* led to little change in HE phase or duty cycle, but saw increased ISF. With relatively high levels of *g_Syn_* (300-600 nS), increasing *Φ_Syn_* (0.2-0.5) caused one HE to decrease its duty cycle while the other increased. Higher levels of *Φ_Syn_* (0.5-0.8) caused the HE’s to switch their relative duty cycle patterns. With weak *g_coup_* (0.25-0.50 nS) and moderate *Φ_Syn_* (0.4-0.6), increasing *g_Syn_* led to a reduced HE duty cycle and side-to-side phase difference. The largest phase differences were found when both *g_Syn_* and *g_coup_* were relatively strong. In summary, increases in *g_coup_* tended to lead to increased phase differences, while increases in *g_Syn_* led to decreased phase differences.

Our search of parameter space has provided a foundation for understanding the mechanisms underlying variable phase differences in neuronal networks and reinforced the importance of compromise between synaptic and neuronal properties for producing functional motor patterns.

## References

[B1] Norris BJ Weaver AL Wenning A Garcia PS Calabrese RL A central pattern generator producing alternative outputs: phase relations of leech heart motor neurons with respect to premotor synaptic input J Neurophysiol 2007 98 5 2983 2991 10.1152/jn.00407.2007 17728387

[B2] García PS Wright TM Cunningham IR Calabrese RL Using a model to assess the role of the spatiotemporal pattern of inhibitory input and intrasegmental electrical coupling in the intersegmental and side-to-side coordination of motor neurons by the leech heartbeat central pattern generator J Neurophysiol 2008 100 3 1354 1371 10.1152/jn.90579.2008 18579654 PMC2544471

[B3] Weaver AL Roffman RC Norris BJ Calabrese RL A role for compromise: synaptic inhibition & electrical coupling interact to control phasing in the leech heartbeat CPG Front Behav Neurosci 2010 4 1 17 20700387 10.3389/fnbeh.2010.00038 PMC2914584

